# Silver nanoparticles induce stress-associated upregulation of VEGF and FGF in fibroblasts as a compensatory response

**DOI:** 10.7717/peerj.21694

**Published:** 2026-09-14

**Authors:** Panida Tepchalee, Pishyaporn Sritangos, Kiattisak Batsungnoen, Nuannoi Chudapongse, Oratai Weeranantanapan

**Affiliations:** 1 School of Preclinical Sciences, Institute of Science, Suranaree University of Technology, Nakhon Ratchasima, Thailand; 2 School of Occupational Health and Safety, Institute of Public Health, Suranaree University of Technology, Nakhon Ratchasima, Thailand; 3 Faculty of Medicine, Assumption University, Samut Prakan, Thailand

**Keywords:** Silver nanoparticles, Fibroblasts, VEGF, FGF, Tissue regeneration, Cell migration, Nanotoxicity, Compensatory response

## Abstract

**Background:**

Silver nanoparticles (AgNPs) are widely incorporated into wound dressings and tissue-engineering scaffolds due to their antimicrobial properties; however, their direct effects on fibroblast biology and angiogenic signaling remain incompletely understood. Fibroblasts play a key role in tissue repair through secretion of vascular endothelial growth factor (VEGF) and fibroblast growth factor (FGF). Elucidating how AgNPs influence fibroblast stress response and growth factor expression is critical for the safe design of regenerative biomaterials.

**Methods:**

Commercially standardized AgNPs were characterized by transmission electron microscopy (TEM) to determine particle size and dispersion. Mouse NIH/3T3 fibroblasts were exposed to various concentrations of AgNPs. Intracellular AgNP localization was visualized by TEM. Reactive oxygen species (ROS) generation was evaluated at 6 h using a dichloro-dihydro-fluorescein diacetate (DCFH-DA)-based assay, with additional cell-free controls to assess nanoparticle–probe interactions, including potential fluorescence quenching effects. Cell viability and migration were evaluated at 24 h using PrestoBlue and wound healing assays, respectively. VEGF and FGF protein expression were quantified by Western blot after 24 h of exposure.

**Results:**

AgNPs with an average diameter of 27.35 ± 11.92 nm were effectively internalized and accumulated in nuclei, perinuclear regions, mitochondria, and cytoplasmic vacuoles. At 6 h, ROS levels showed an increasing trend with AgNP exposure, although not statistically significant. Cell-free experiments demonstrated a dose-dependent reduction in DCF fluorescence in the presence of AgNPs, confirming nanoparticle-induced quenching and suggesting that intracellular ROS levels may be underestimated. At 24 h, AgNPs significantly reduced cell viability at 100 µg/mL, while migration exhibited a non-significant increase at 50 µg/mL. Despite these stress responses, VEGF and FGF levels increased significantly in a concentration-dependent manner, reaching statistical significance at the highest concentration.

**Conclusion:**

AgNP exposure induces intracellular accumulation and early oxidative stress, followed by upregulation of VEGF and FGF in fibroblasts. However, the absence of corresponding functional enhancement in cell migration, together with cytotoxic effects at higher concentrations, indicates that this response reflects a compensatory stress-associated mechanism rather than functional angiogenesis. These findings highlight the importance of validating nanoparticle–assay interactions and emphasize the need for careful dose optimization in the development of AgNP-based regenerative biomaterials.

## Introduction

Silver nanoparticles (AgNPs) have emerged as one of the most extensively studied and utilized nanomaterials in modern biomedical and industrial applications. Their remarkable antimicrobial activity, high surface-area-to-volume ratio, electrical conductivity, and chemical stability have led to their incorporation into a wide range of products, including food storage, textile coating, medical device coatings, tissue regeneration materials (Pannerselvam et al., 2017; Burduşel et al., 2018; Kumar et al., 2018; Zhou & Tang, 2018; Xu et al., 2020; Sati et al., 2025). Due to their nano-scale dimensions and biological reactivity, AgNPs can interact closely with cellular structures and molecular pathways, exerting both therapeutic and toxicological effects depending on size and shape, concentration and exposure time, surface change, and cell type involved (Xu et al., 2020).

The increasing use of AgNPs in clinical and biomedical settings has raised safety concerns regarding their biocompatibility and potential long-term effects on human cells and tissues. Among the various cell types exposed to these materials, fibroblasts are of particular interest due to their central role in tissue homeostasis, extracellular matrix (ECM) production, wound healing, and tissue regeneration (Tracy, Minasian & Caterson, 2016). NIH/3T3 fibroblasts, derived from mouse embryonic tissue, are widely used as a model system to evaluate cytotoxicity, proliferation, migration, and signaling responses to materials intended for tissue engineering and regenerative medicine. Understanding how AgNPs influence fibroblast behavior at both cellular and molecular levels is therefore critical for the safe and effective development of nanoparticle-integrated biomedical devices.

Previous studies have consistently shown that AgNPs exert dose-dependent cytotoxic effects on mammalian cells through mechanisms involving oxidative stress, mitochondrial dysfunction, and DNA damage (Chairuangkitti et al., 2013; Wang et al., 2017; Li et al., 2020). However, recent research has also highlighted the complexity of cellular responses to AgNPs, exhibiting dual actions, inducing stress responses while simultaneously activating signaling pathways related to cell repair or adaptation (Palanisamy et al., 2024; Sati et al., 2025). Such stress-associated responses may include alterations in the expression of growth factors and cytokines, even under conditions where overall cellular function is compromised (Löfdahl et al., 2020).

Angiogenesis, the process of new blood vessel formation, is essential for wound healing, tissue repair, and integration of implanted biomaterials (Veith et al., 2019; Wang et al., 2024). Although angiogenesis-related factors such as VEGF and FGF are classically associated with tissue repair and neovascularization, their upregulation may also reflect a stress-adaptive response and therefore does not necessarily indicate functional angiogenic activation (Kim & Byzova, 2014; Smith et al., 2015). Fibroblasts contribute to tissue repair not only through ECM remodeling but also *via* paracrine signaling, including the secretion of VEGF and FGF, which help establish a pro-angiogenic microenvironment and modulate the behavior of surrounding cells (Cialdai, Risaliti & Monici, 2022; Zhang et al., 2023). However, the extent to which AgNP-induced changes in these growth factors reflect adaptive stress signaling, rather than enhanced regenerative function, remain unclear. This distinction is particularly critical in biomaterials research, where increased expression of pro-angiogenic factors is often interpreted as a beneficial outcome without sufficient consideration of underlying cellular stress. While several studies reported that certain forms of cellular stress, including AgNP exposure, can alter the expression of pro-angiogenic factors (Sheikpranbabu et al., 2009; Franková et al., 2016; Palanisamy et al., 2024), it remains unclear whether these responses correspond to improved cellular function or instead represent compensatory mechanisms triggered by nanoparticle-induced stress.

The influence of AgNPs on fibroblast behavior is particularly relevant in tissue engineering applications, where biomaterials must not only avoid cytotoxicity but also preserve essential cellular functions such as migration, paracrine signaling, and ECM remodeling. Accordingly, distinguishing between functional activation and stress-associated signaling is critical for the rational design of AgNP-containing biomaterials.

In this study, we hypothesized that exposure to standardized, commercially sourced AgNPs induces cellular stress responses in fibroblasts, accompanied by alterations in growth factor expression. Specifically, we aimed to determine whether AgNP exposure within a concentration range relevant to biomaterial–cell interface exposure induces cytotoxicity and modulates VEGF and FGF expression, and whether these molecular changes are associated with corresponding functional outcomes. To address this, cytotoxicity, intracellular localization, early oxidative responses, migration, and pro-angiogenic protein expression were evaluated in NIH/3T3 fibroblasts following AgNP exposure. Collectively, this work seeks to clarify whether AgNP-induced growth factor upregulation reflects functional pro-angiogenic activity or a stress-associated compensatory response, highlighting a potential disconnect between molecular signaling and cellular function.

## Materials & Methods

### Chemicals and reagents

Silver nanopowder was purchased from Sigma-Aldrich (St. Louis, MO, USA). Dulbecco’s Modified Eagle Medium (DMEM), trypsin, penicillin-streptomycin, and fetal bovine serum (FBS) were obtained from Gibco (New York, NY, USA). PrestoBlue™ Cell Viability Reagent and Actin Recombinant Rabbit Monoclonal Antibody (JJ09-29) were sourced from Invitrogen (Carlsbad, CA, USA). Anti-VEGF antibody (ab46154) and anti-FGF21 antibody (ab171941) were procured from Abcam (Cambridge, UK). 10X Tris-Glycine/SDS and Precision Plus Protein™ Dual Xtra Standards were purchased from Bio-Rad Laboratories (Hercules, CA, USA). All other chemicals and reagents used in this study were of analytical grade.

### Particle preparation

AgNPs were weighed using a high-precision microbalance, and a stock suspension was prepared at a concentration of 10 mg/mL in absolute ethanol. These suspensions are sonicated overnight in a water bath sonicator, sterilized with ultraviolet (UV) light for 30 min and kept in stock at 4 °C until use.

### Cell culture and AgNP treatment

NIH/3T3 mouse fibroblast cells (CRL-1658) were obtained from the American Type Culture Collection (ATCC, Manassas, VA, USA). Cells were cultured in Dulbecco’s Modified Eagle Medium (DMEM) supplemented with 10% (v/v) fetal bovine serum (FBS) and 1% (v/v) penicillin-streptomycin. Cultures were maintained in T75 flasks at 37 °C in a humidified incubator with 5% CO_2_. The culture medium was replaced every 2–3 days, and cells were passaged at 70–80% confluency using 0.25% trypsin-Ethylenediaminetetraacetic Acid (EDTA).

For experiments, cells were seeded at indicated densities depending on the assay and allowed to adhere overnight prior to treatment. AgNPs were dispersed in culture medium and applied at final concentrations of 0, 25, 50, and 100 μg/mL for the indicated time points. This concentration range was selected based on preliminary cytotoxicity assessment.

### Characterization of AgNPs

The morphology, size, and shape of AgNPs were characterized using transmission electron microscopy (FE/TEM; Thermo Scientific TALOS F200X, Waltham, MA, USA) under both pre- and post-sonication conditions. Particle size distribution was determined from seven representative TEM images comprising 182 particles, acquired at 9400× magnification with a 100 nm scale bar, and analyzed using ImageJ software. Dynamic light scattering (DLS; Malvern Zetasizer Nano-ZS, UK) was employed to assess hydrodynamic diameter and dispersity. For DLS measurements, AgNPs were prepared as a stock suspension (10 mg/mL) in deionized water, sonicated for 24 h, and subsequently diluted 50,000-fold in deionized water prior to analysis.

### Cellular uptake test

NIH/3T3 cells were seeded in T25 flasks at a density of 1 × 10^6^ cells and incubated for 24 h. The cells were then treated with AgNPs at concentrations of 0, 25, and 50 µg/mL for 24 h in serum-free DMEM (0% FBS). After treatment, the cells were trypsinized, collected, and washed twice with 1X phosphate-buffered saline (PBS). The cell pellets were fixed overnight at 4 °C in 2.5% glutaraldehyde, washed with 1X PBS, and processed for ultrastructural analysis of AgNP uptake by TEM.

Osmium tetroxide staining was used to enhance membrane contrast; however, this electron-dense stain may complicate identification of other electron-dense structures. To minimize misinterpretation, AgNPs were identified based on their distinct size, morphology, and electron density, which were consistent with the characterized nanoparticles.

### ROS detection assay

Intracellular reactive oxygen species (ROS) levels were measured using a DCFH-DA-based assay (ab113851, Abcam, Cambridge, UK) according to the manufacturer’s instructions. NIH/3T3 cells were seeded in 96-well plates with clear flat bottom and black sides for 1.5 × 10^4^ cells/well and allowed to adhere for 24 h. Cells were then incubated with 15 μM DCFH-DA in 1X buffer 100 μL/well at 37 °C for 45 min in the dark. After incubation, cells were washed with 1X PBS to remove excess probe. Cells were then treated with AgNPs at final concentrations of 0, 25, 50, and 100 μg/mL in 1X supplemented buffer (100 μL/well) for 6 h. Following treatment, fluorescence intensity was measured using a microplate reader (CLARIO star Plus, Ortenberg, Germany) with excitation/emission wavelengths of 485/535 nm. Data were expressed as a percentage of the untreated control. Tert-butyl hydroperoxide (TBHP) was used as a positive control for ROS generation under the same experiment conditions. All procedures were performed in accordance with the Methods previously described (Satsantitham et al., 2024).

To assess potential nanoparticle–probe interactions, complementary cell-free control experiments were performed. In these experiments, 30 μM DCFH-DA 50 μL/well was first oxidized to its fluorescent form (DCF) using 0.01N sodium hydroxide (NaOH) five μL/well for 30 min. The oxidized probe was then incubated with AgNPs prepared in 1X supplemented buffer (45 μL/well), with or without TBHP, to achieve final AgNPs concentrations of 0, 25, 50, and 100 μg/mL in a total reaction volume of 100 μL/well. The Mixtures were incubated for 6 h. Fluorescence intensity was measured under the same settings as described above to evaluate potential quenching or interference effects of AgNPs on the fluorescent signal.

### Cytotoxicity test

Cell viability was evaluated using the PrestoBlue assay. NIH/3T3 cells were seeded into 96-well plates at a density of 2 × 10^4^ cells per well and incubated for 24 h in DMEM supplemented with 10% FBS. AgNPs were vortexed and diluted from a 10 mg/mL stock solution to final concentrations ranging from 0 to 100 µg/mL. Cells were then treated with AgNPs for 24 h in serum-free DMEM (0% FBS). After treatment, 10 µL of PrestoBlue reagent was added to each well, and optical density (OD) was measured at 570 nm and 600 nm using a microplate reader (Thermo Multiskan FC). To account for potential interference from AgNPs, all data were corrected for background absorbance measured in cell-free wells containing AgNPs. Untreated cells served as the 100% viability control. In addition, changes in cell morphology following AgNP exposure were examined using an inverted microscope (Olympus CKX41, Tokyo, Japan).

### Wound healing assay

Cell migration was evaluated using a wound healing assay. NIH/3T3 cells were seeded into Ibidi Culture-Inserts (Culture-Insert 2 Well, Cat. No. 80209, Ibidi, Gräfelfing, Germany) placed in 24-well culture plates. A cell suspension (1 × 10^5^ cells/mL) was prepared in DMEM supplemented with 10% fetal bovine serum (FBS), and 70 μL of the suspension was added to each reservoir of the insert. Cells were allowed to adhere and form a confluent monolayer over 24 h. After incubation, the Culture-Inserts were carefully removed to create a defined cell-free gap. The cells were gently washed with 1X PBS to remove detached cells and debris. Cells were then treated with AgNPs at concentrations of 0 or 50 μg/mL in serum-free DMEM (500 μL/well) for 24 h. The concentration of 50 μg/mL was selected as the highest dose that did not induce significant cytotoxicity based on preliminary viability assessment. Phase-contrast images were captured at 0, 3, 6, and 24 h from the same fields of view using a phase-contrast microscope. For quantitative analysis, three images per condition were analyzed from three independent experiments. The wound area was measured using ImageJ software. Gap closure due to cell migration was calculated based on changes in wound area over time and expressed as a percentage of wound closure relative to the initial gap area, using the following formula:

\begin{eqnarray*}\%\text{ wound closure}= \frac{\text{Initial area}-\text{Current area}}{\text{Initial area}} \times 100. \end{eqnarray*}

### Western blot assay

Growth factor production in NIH/3T3 cells was assessed using Western blotting. Cells were seeded at a density of 2 × 10^6^ cells/mL and exposed to AgNP-treated or untreated conditions for 24 h. Following treatment, cells were harvested and lysed in radioimmunoprecipitation assay (RIPA) buffer. Equal amounts of denatured protein (30 µg) were separated on a 12% SDS-polyacrylamide gel and transferred onto nitrocellulose membranes. To block non-specific binding, membranes were incubated with 5% skimmed milk for 1 h, followed by overnight incubation at 4 °C with primary antibodies against VEGF and FGF (1:1000 dilution). After washing, membranes were incubated with HRP-conjugated anti-rabbit secondary antibodies (1:2000 dilution) for 2 h at room temperature. *β*-Actin (1:4000 dilution) was used as an internal loading control. Target protein bands were visualized using enhanced chemiluminescence (Amersham ImageQuant™ 500, USA) and quantified by densitometric analysis with ImageJ software.

### Statistical analysis

All experiments were performed in triplicate. Data are presented as the mean ± standard deviation (SD) or the mean ± standard error of the mean (SEM). Normality was confirmed using a standard test, and datasets were normally distributed. An unpaired *t*-test was applied for two-group comparisons, while one-way analysis of variance (ANOVA) followed by Dunnett’s *post hoc* test was conducted for multiple-group comparisons. Statistical analyses were performed with GraphPad Prism software, version 10.2.3 (GraphPad Software, Inc.). A *p*-value < 0.05 was considered statistically significant.

## Results

### Characterization of AgNPs

**Figure 1:**
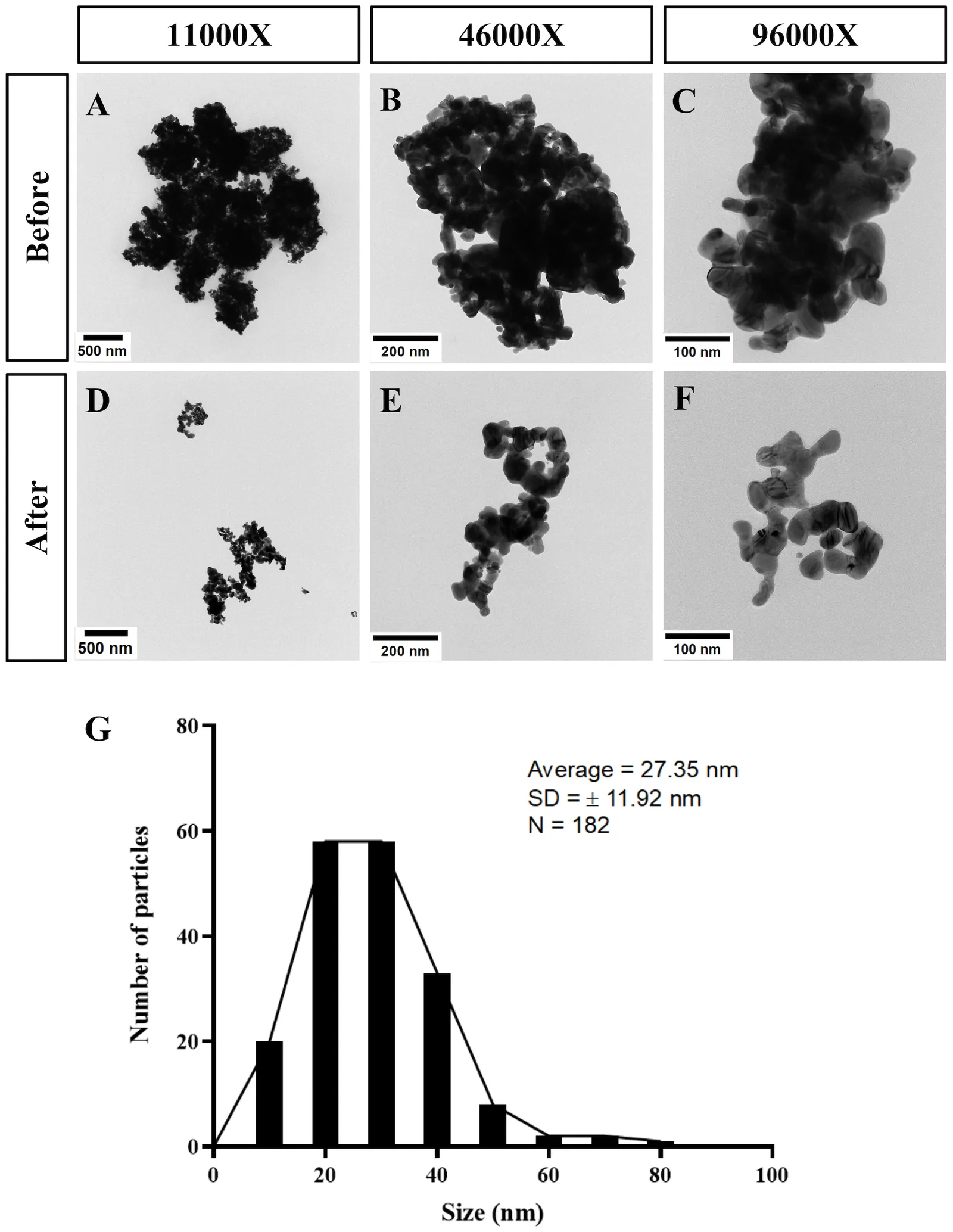
Characterization of AgNPs by TEM. Representative TEM images of AgNPs before (A–C) and after (D–F) sonication are shown. The images demonstrate particle morphology and dispersion under both conditions. (G) Histogram showing the particle size distribution of AgNPs, with an average diameter of 27.35 ± 11.92 nm.

Because nanoparticle dispersion and aggregation critically influence cellular interactions, TEM characterization was performed following sample preparation to confirm the physicochemical state of AgNPs under the experimental conditions used for subsequent biological assays (Fig. 1). TEM analysis revealed that the AgNPs exhibited a predominantly spherical morphology. Following 24 h of sonication, a more uniform particle distribution was observed (Figs. 1D–1F), indicating improved dispersion of the nanoparticles under the preparation conditions used in this study.

Dynamic light scattering (DLS) analysis further characterized the particle size distribution, revealing a polydispersity index (PDI) of 0.644 and a Z-average hydrodynamic diameter of 643.30 ± 214.45 nm, suggesting some degree of agglomeration in aqueous suspension. To verify primary size, TEM images were quantified using Image J software. As shown in Fig. 1G, the average particle diameter was 27.35 ± 11.92 nm (range: 5.43–81.47 nm), with over 80% of the particles falling within 20–40 nm range, indicating a relatively broad but nanoscale distribution.

### Intracellular localization of AgNPs in NIH/3T3 cells

**Figure 2:**
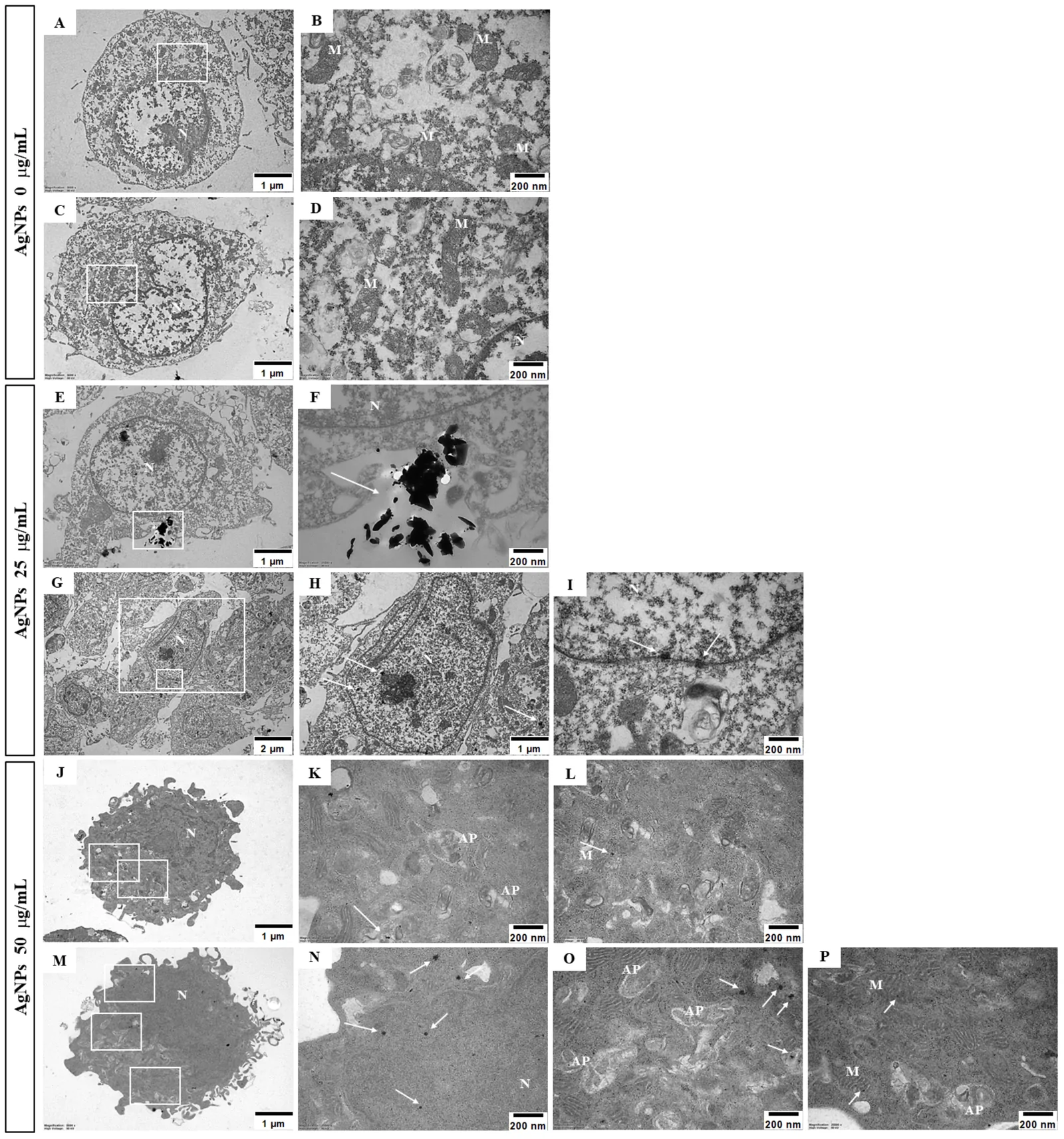
Intracellular localization of AgNPs in NIH/3T3 cells. NIH/3T3 cells were treated with 0, 25, and 50 µg/mL AgNPs for 24 h and analyzed by TEM. (A–D) Representative images of untreated cells (0 µg/mL). (E–I) Cells treated with 25 µg/mL AgNPs, showing nanoparticle internalization within the cytoplasm, nucleus, and other intracellular compartments. (J–P) Cells treated with 50 µg/mL AgNPs, exhibiting increased intracellular accumulation along with morphological alterations, including membrane blebbing, cell shrinkage, and autophagic (AP) vesicle-like structures. AgNPs were observed in the nucleus (N), mitochondria (M), and cytoplasmic vacuoles. Scale bars: one µm in (A), (C), (E), (H), (J), and (M); two µm in (G); and 200 nm in (B), (D), (F), (I), (K), (L), (N), (O), and (P).

To investigate the intracellular localization of AgNPs in NIH/3T3 cells, TEM analysis was performed following exposure to selected concentrations. As shown in Fig. 2, AgNPs exhibited a concentration-dependent pattern of intracellular accumulation. Even at the lowest tested concentration (25 μg/mL), AgNPs were detected in the nucleus, perinuclear region, mitochondria, and vacuoles (Figs. 2F, 2H, and 2I). At 50 μg/mL, AgNPs induced marked morphological alterations, including blebbing, shrinkage, and the presence of autophagic vesicles (Figs. 2K, 2L, 2O and 2P), indicating cellular stress and structural damage. Notably, the localization of AgNPs within mitochondria (Fig. 2P) suggests a potential link to oxidative stress-related mechanisms.

### Early oxidative stress response and AgNP-probe interaction

**Figure 3:**
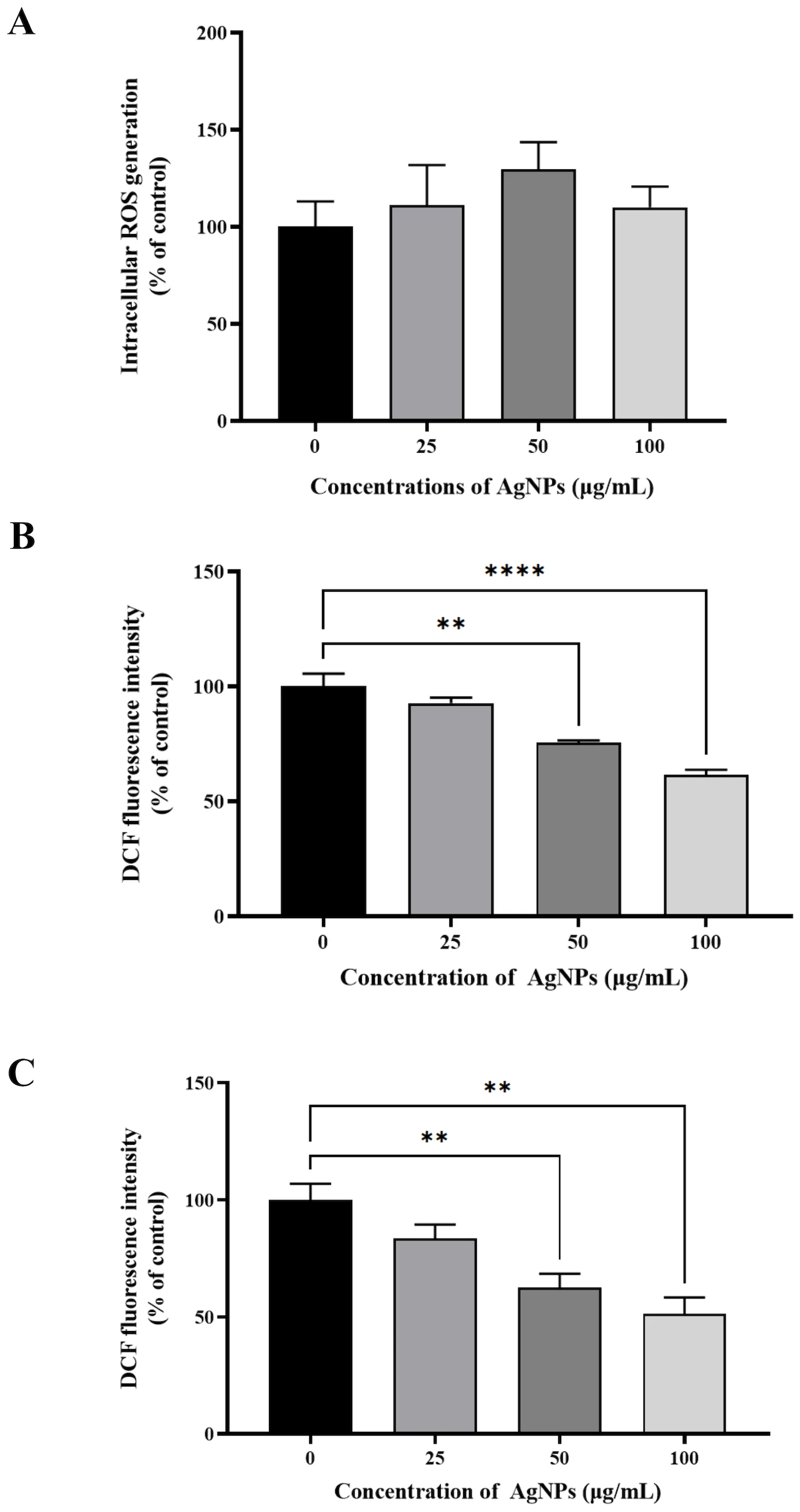
Early oxidative stress response and assessment of AgNPs–probe interactions. Intracellular reactive oxygen species (ROS) generation and potential AgNPs–probe interactions were evaluated following AgNP exposure. (A) Intracellular ROS levels in NIH/3T3 cells after 6 h treatment with 0–100 µg/mL of AgNPs, measured using a DCFH-DA-based assay. Data are expressed as a percentage of the untreated control. Statistical significance was determined by one-way ANOVA. No significant difference was observed compared with untreated control (*p* > 0.05). (B) Cell-free system containing oxidized probe (DCF), TBHP, and AgNPs, demonstrating the effect of AgNPs on fluorescence intensity under oxidative conditions. (C) Cell-free system containing oxidized probe (DCF) and AgNPs in the absence of TBHP, assessing direct nanoparticle–dye interactions. A dose-dependent reduction in fluorescence intensity was observed in both cell-free conditions (B and C), indicating nanoparticle-induced quenching of DCF signal. All data are presented as mean ± SEM from three independent experiments. Statistical significance compared to the untreated control is indicated as **p < 0.05 and ****p < 0.0001.

To further investigate the possible involvement of mitochondrial localization of AgNPs in oxidative stress, intracellular ROS levels were assessed after 6 h of exposure. As shown in Fig. 3A, AgNP treatment resulted in an increase in ROS levels compared with the untreated control; however, this increase did not reach statistical significance. To determine whether AgNPs interfered with fluorescence-based ROS detection, cell-free control experiments were performed. As shown in Fig. 3B, in the presence of TBHP and oxidized probe (DCF), AgNPs induced a concentration-dependent reduction in fluorescence intensity, which was statistically significant at 50 and 100 μg/mL (*p* = 0.0019 and *p* < 0.0001, respectively). A similar dose-dependent decrease in fluorescent intensity was observed in the absence of TBHP, with significant reductions also detected at 50 and 100 μg/mL (*p* = 0.0090 and *p* = 0.0019, respectively) (Fig. 3C). These cell-free control findings indicate that AgNPs directly affect the fluorescent signal of the probe. Together, the results suggested that AgNPs can reduce DCF fluorescence intensity under cell-free conditions, which may lead to an underestimation of intracellular ROS levels, particularly at higher nanoparticle concentrations.

### Cytotoxic effects of AgNPs on NIH/3T3 cells

**Figure 4:**
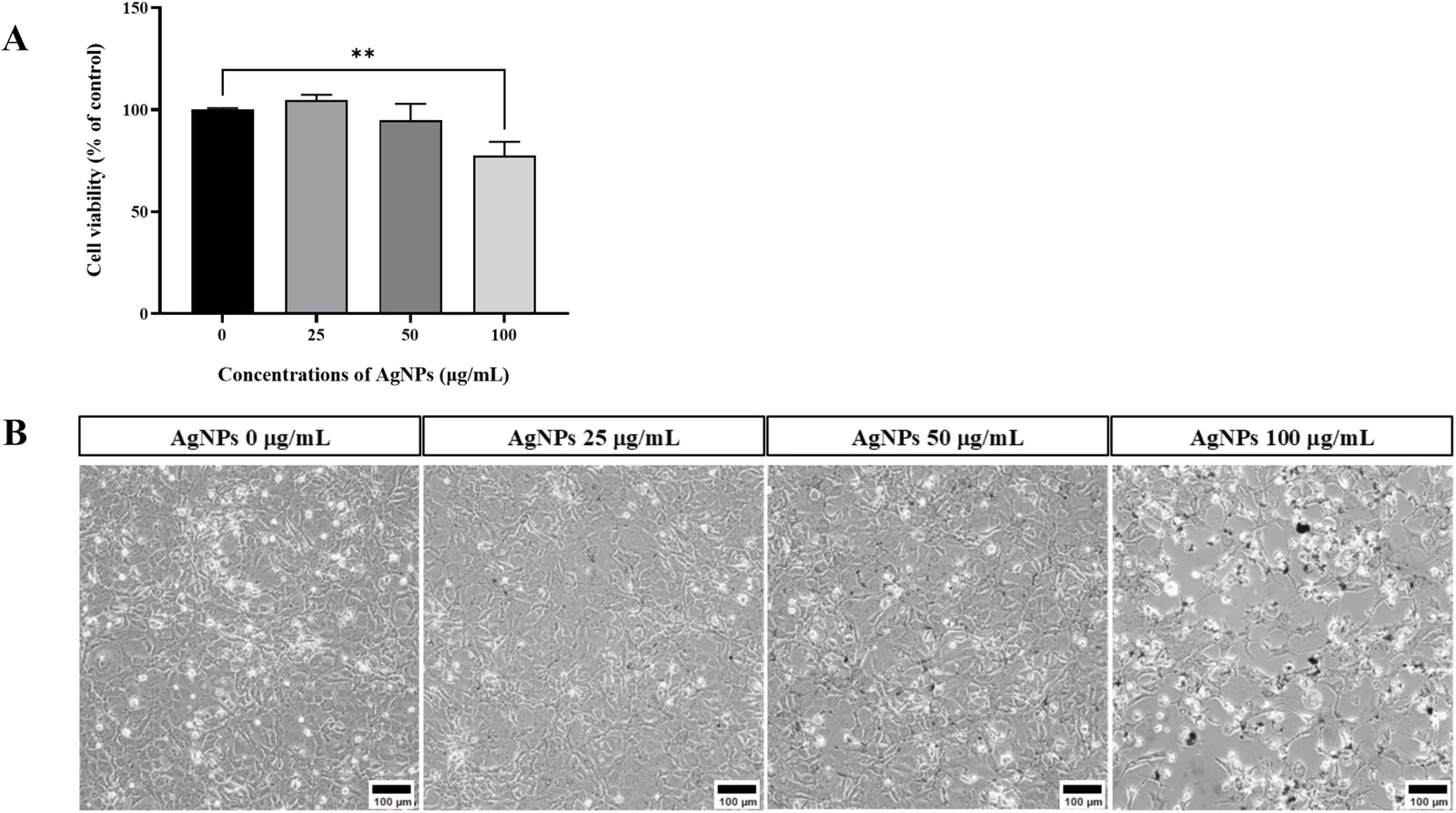
Cytotoxic effects of AgNPs on NIH/3T3 cells. NIH/3T3 cells were treated with 0, 25, 50, and 100 µg/mL AgNPs for 24 h. Cell viability was assessed using the PrestoBlue assay. (A) Cell viability expressed as a percentage of the untreated control. Data represent mean ± SEM from three independent experiments. **p < 0.01 compared with the control group. (B) Representative images showing morphological changes in AgNP-treated NIH/3T3 cells after 24 h. Scale bar: 100 µm.

Following the assessment of early oxidative stress responses, the cytotoxic effects of AgNPs on NIH/3T3 cells was evaluated after 24 h of exposure using the PrestoBlue assay. As shown in Fig. 4A, exposure to 100 μg/mL AgNPs significantly reduced cell viability to 77.44 ± 6.81% compared with untreated controls (*p* = 0.0026), while lower concentrations showed no significant effect. Consistent with these findings, morphological changes were observed (Fig. 4B), including cell shrinkage, detachment, and intracellular AgNP accumulation. These results indicated that higher concentrations of AgNPs induce measureable cytotoxic effects in NIH/3T3 cells.

### Effect of AgNPs on NIH/3T3 cell migration

**Figure 5:**
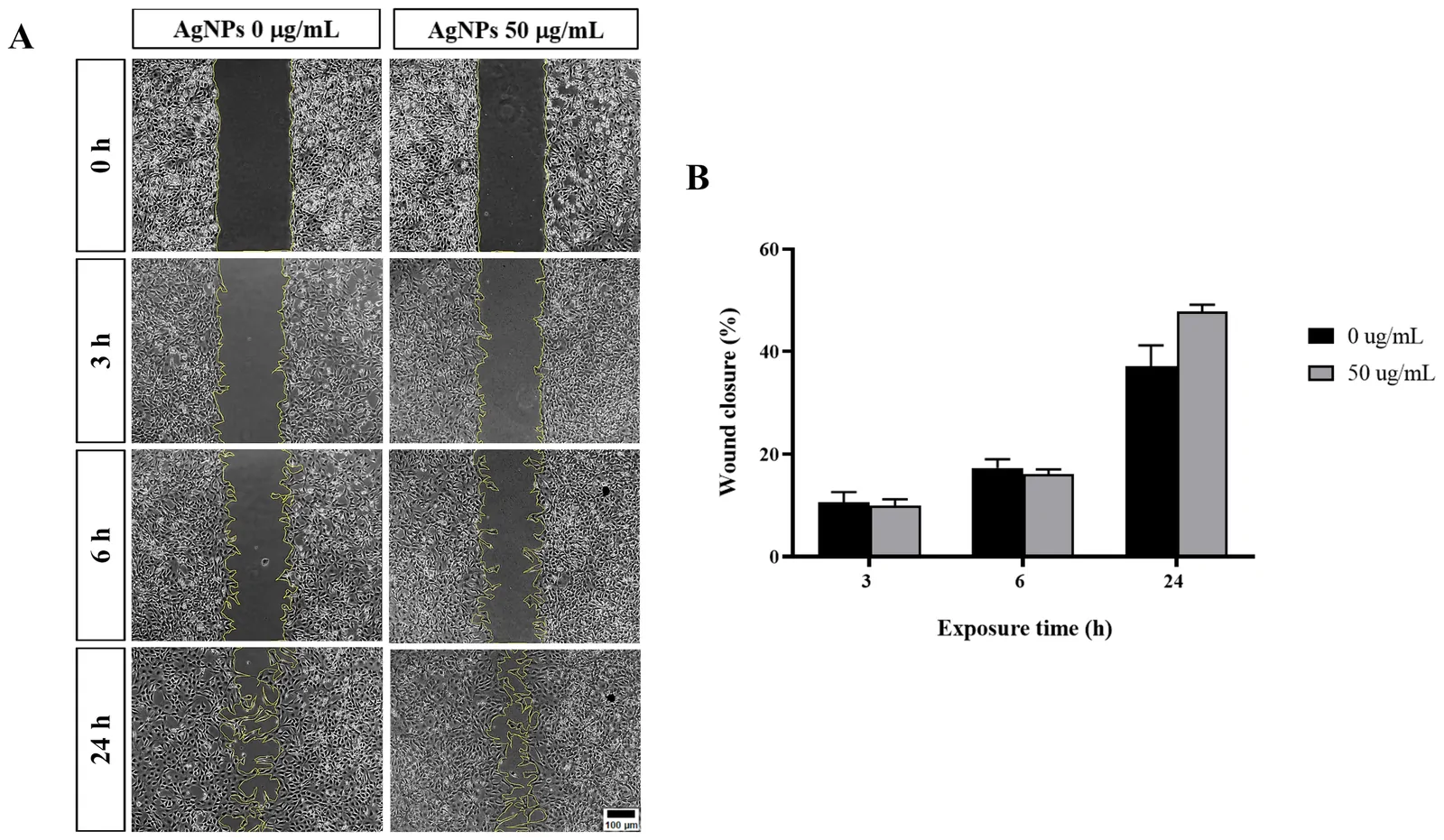
Effects of AgNPs on the migration of NIH/3T3 cells. NIH/3T3 cell migration was examined by wound healing assay. The cells were treated with a concentration of 50 μg/mL of AgNPs in DMEM serum-free medium. (A) Representative images of the wound healing assay at 0, 3, 6, and 24 h. (B) Quantitative analysis of the percentage of wound closure. Data are presented as mean ± SEM from three independent experiments. No significant difference was observed compared to 24 h untreated control (*p* > 0.05). Scale bar: 100 mm.

Because migration of fibroblasts is an important process of tissue regeneration, the effect of AgNPs on NIH/3T3 cell migratory capacity was evaluated using a wound healing assay at a non-toxicity concentration. As shown in Fig. 5, exposure to 50 μg/mL AgNPs for 24 h resulted in a slight increase in cell migration compared with the untreated control; however, this effect was not statistically significant. These findings indicated that AgNP exposure does not significantly enhance fibroblast migratory capacity under the tested conditions.

### Effect of AgNPs on VEGF and FGF protein expression in NIH/3T3 cells

**Figure 6:**
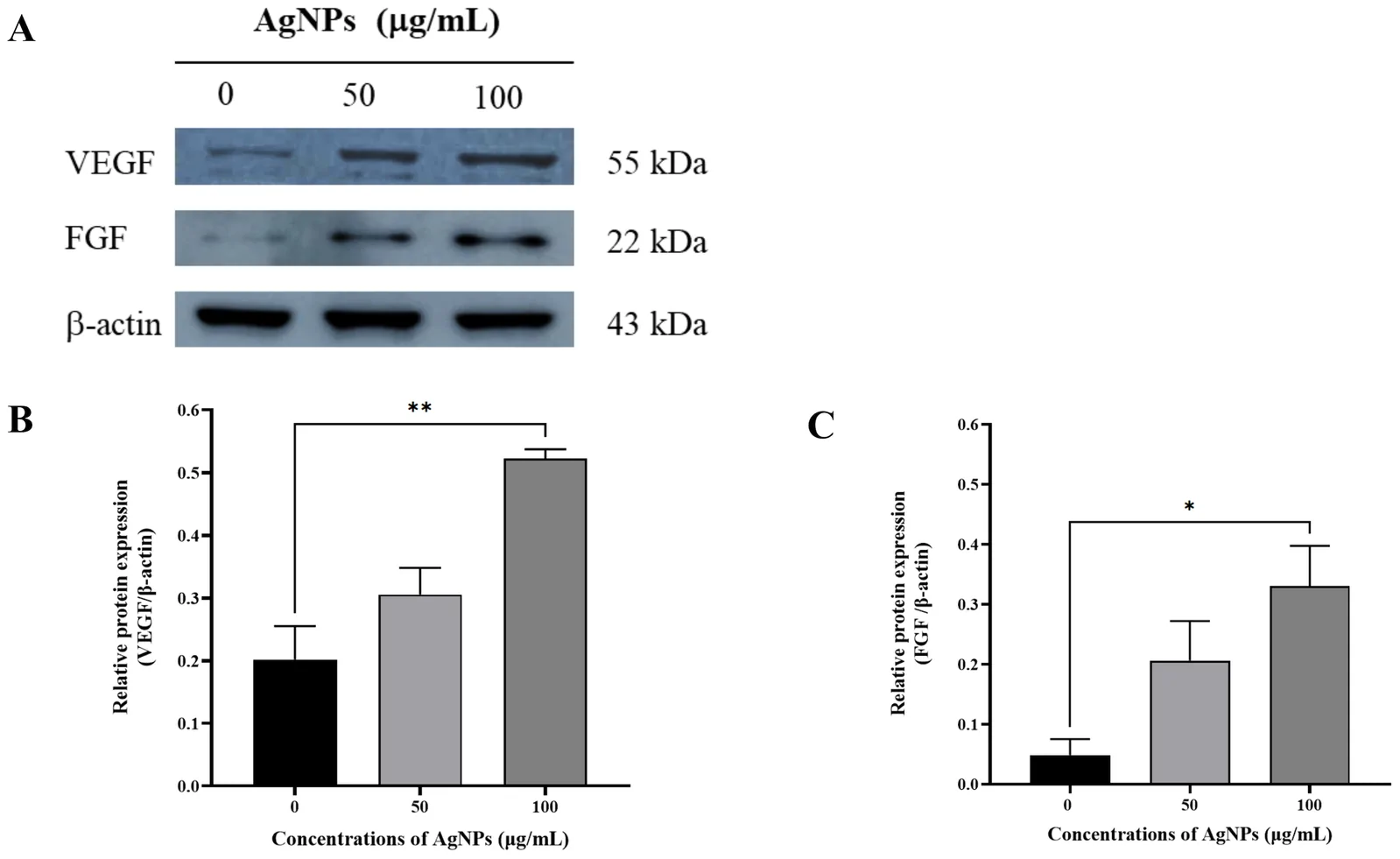
Effects of AgNPs on the protein expression levels of VEGF and FGF in NIH/3T3 fibroblast cells. (A) Representative Western blot bands showing VEGF and FGF expression in the NIH/3T3 cells treated with different concentrations of AgNPs for 24 h. Quantitative analysis of (B) VEGF and (C) FGF protein expression based on densitometry of Western blots. Data were obtained by using Image J software expressed as the mean ± SEM (*n* = 3). *p < 0.05 and **p < 0.01 compared with untreated control.

Angiogenesis-associated growth factors, including VEGF and FGF, play key roles in neovascularization by supporting the delivery of oxygen and nutrients during tissue repair. Following the assessment of early oxidative stress responses, the association between AgNP exposure and angiogenesis-associated protein expression was evaluated. VEGF and FGF protein levels were assessed in NIH/3T3 fibroblasts after 24 h of treatment using Western blot analysis. As shown in Fig. 6, both factors were upregulated in a dose-dependent manner, with the most pronounced and statistically significant increases observed at 100 µg/mL (*p* = 0.0032, *p* = 0.0283, respectively). Collectively, these findings revealed a molecular-functional disconnect in fibroblast responses to AgNP exposure, linking stress-associated signaling with limited functional outcomes.

## Discussion

AgNPs are increasingly incorporated into wound dressings and regenerative biomaterials for their potent antimicrobial activity, yet their cellular impacts on fibroblast function remain incompletely characterized, particularly in relation to stress responses and angiogenic signaling. Fibroblasts are critical for tissue repair, coordinating extracellular matrix remodeling and the release of paracrine factors. In the present study, exposure of NIH/3T3 fibroblasts to commercially sourced AgNPs resulted in intracellular nanoparticle accumulation and concentration-dependent cytotoxicity, accompanied by early oxidative response, although the increase in ROS did not reach statistical significance. TEM analysis revealed extensive intracellular accumulation of AgNPs. Functionally, AgNP exposure led to a modest increase in fibroblast migration; however, this effect was not statistically significant. Despite these indicators of cellular stress, AgNP treatment induced a significant upregulation of the angiogenesis-associated growth factors, VEGF and FGF, suggesting activation of stress-associated signaling pathways rather than a corresponding enhancement of functional repair capacity.

TEM analysis revealed that the AgNPs exhibited a predominantly spherical morphology, consistent with previous reports using the same commercial product (Chairuangkitti et al., 2013; Xin et al., 2015; Szymczak et al., 2024). Regarding the size distribution results obtained from DLS, the relatively high standard error of the mean reflected the wide range of particle sizes present, consistent with the broad distribution detected by DLS. Prior work using the same commercial AgNPs also reported differences in hydrodynamic size, likely attributable to sample preparation and aggregation (Chairuangkitti et al., 2013). A relatively high PDI is often associated with particle aggregation or the coexistence of distinct subpopulations of particle sizes (Szymczak et al., 2024). Most particles measured by TEM fell within the 20–40 nm range, in agreement with earlier findings for this same commercial AgNPs (Chairuangkitti et al., 2013). Although TEM confirmed the nanoscale core size, the hydrodynamic diameters obtained by DLS were substantially larger. Such differences are well documented, as DLS captures the hydrated particle diameter in suspension, including solvation layers and aggregates, whereas TEM measures the dry physical core size (Domingos, Tufenkji & Wilkinson, 2009; Mourdikoudis, Pallares & Thanh, 2018).

The cytotoxic effects of AgNPs observed in NIH/3T3 fibroblasts are consistent with previous reports of AgNP toxicity in both fibroblastic and non-fibroblastic cell types. The same commercial AgNPs have been shown to induce dose-dependent cytotoxicity and morphological alterations in A549 lung carcinoma cells (Chairuangkitti et al., 2013). Similarly, synthesized spherical AgNPs of comparable size reduced the viability of primary mouse fibroblasts with an IC_5__0_ of 61 µg/mL (Arora et al., 2009). Another study demonstrated that similar sized of synthesized AgNPs caused dose-dependent loss of viability in NIH/3T3 cells (Gurunathan et al., 2018). Even at concentrations as low as 10 µg/mL, AgNPs of similar average size but narrower PDI produced notable morphological changes in NIH/3T3 cells after 24 h of exposure (Lee et al., 2014). The magnitude of toxicity has been closely linked to particle size and dispersion. Smaller, less-stable AgNP agglomerates suspended in biological media generally exhibit greater cytotoxicity than larger, more aggregated particles (Ha, Shim & Yoon, 2018). The presence of a substantial fraction of small particles in the current preparation likely contributed to the observed cytotoxic response. Indeed, AgNPs smaller than 20 nm have been reported to produce stronger toxic effects on human periodontal fibroblasts (Hernández-Sierra et al., 2011) and normal human dermal fibroblasts (Avalos et al., 2016) than larger counterparts. Taken together, these results suggest that the cytotoxicity observed in NIH/3T3 cells is primarily associated with the smaller-sized fraction of AgNPs, which enhances cellular uptake and contributes to the observed morphological alterations.

Our ultrastructural analysis confirmed intracellular localization of AgNPs, in agreement with studies showing that spherical AgNPs can enter cells through multiple pathways and accumulate in various organelles (Wu et al., 2019). For example, the study of synthesized AgNPs in human mesenchymal stem cells demonstrated that they were internalized *via* clathrin-dependent endocytosis and macropinocytosis, subsequently localizing in the perinuclear region (Greulich et al., 2011). Smaller particles, approximately five nm in diameter, have been shown to penetrate more readily and distribute widely across organelles (Liu et al., 2010; Wu et al., 2019). Such accumulation has been associated with mitochondrial dysfunction, swelling, elevated reactive oxygen species (ROS), loss of mitochondrial membrane potential, and apoptosis through the mitochondrial pathway (AshaRani et al., 2009; Chairuangkitti et al., 2013; Xue et al., 2018; Li et al., 2020). Smaller AgNPs also achieve higher uptake into cytoplasmic vesicles, promoting autophagy and lysosomal activation (Mishra et al., 2016). Consistent with these mechanisms, autophagic vesicles were evident in the current study, aligning with reports of AgNP-induced autophagosome formation, increased cytoplasmic vesicles, and mitochondrial alterations in fibroblasts (Lee et al., 2014; Gurunathan et al., 2018). Collectively, the presence of autophagic vesicles may represent an early stress response that contributes to the more pronounced cytotoxicity observed at 100 µg/mL. Nevertheless, it is important to note that our ultrastructural observations remain qualitative due to the limited number of high-resolution TEM sections available for comprehensive statistical counting. Furthermore, the tendency of AgNPs to form dense clusters within cellular compartments, potentially exacerbated by dehydration and resin embedding during sample processing, hindered the precise quantification of individual particles. While electron-dense precipitates from osmium tetroxide staining can occasionally mimic small nanoparticles, we differentiated the internalized AgNPs based on their distinct spherical morphology and localization within endosomal structures. Future studies utilizing ICP-MS will be necessary to provide more rigorous quantitative estimates of cellular uptake to complement these qualitative findings.

The interpretation of ROS measurements in nanoparticle studies is inherently complex due to potential nanoparticle–probe interactions. In the present study, although a trend toward increased ROS levels was observed, this change did not reach statistical significance. Notably, cell-free experiments demonstrated a concentration-dependent reduction in DCF fluorescence intensity in the presence of AgNPs, indicating direct interference with the probe signal. This observation is consistent with previous studies showing that nanoparticles can interact with DCFH-DA-based assays, leading to fluorescence quenching and masking of oxidative signals (Aranda et al., 2013). Importantly, engineered nanomaterials have been systematically shown to interfere with acellular DCFH assays, potentially generating misleading oxidative readouts through surface reactivity and optical effects (Kroll et al., 2012; Pal et al., 2012; Roesslein et al., 2013). Consistent with the present findings, nanoparticles have been reported to induce concentration-dependent quenching of DCF fluoresce under cell-free conditions (Roesslein et al., 2013). More broadly, nanoparticle interference with assay components has been widely reported to result in both over- and underestimation of biological responses, depending on nanoparticle properties and assay conditions (Ong et al., 2014). Notably, previous work using the same commercial AgNPs in A549 cells demonstrated that AgNP-induced cytotoxicity was at least partly associated with intracellular ROS generation, accompanied by reduced cell viability and mitochondrial membrane potential (MMP) (Chairuangkitti et al., 2013). These findings support the involvement of oxidative stress as a contributing mechanism of AgNP toxicity. In this context, the modest and non-significant ROS increase observed in the present study may not necessarily indicate the absence of oxidative stress, but rather reflect differences in cell type, experimental conditions, or technical limitations of the assay, including nanoparticle-induced quenching effects. Taken together, these findings suggest that the ROS response to AgNP exposure should be interpreted with caution, as fluorescence-based measurements may underestimate intracellular oxidative stress, particularly at higher nanoparticle concentrations. These considerations underscore the importance of incorporating cell-free controls and complementary approaches when evaluating nanoparticle-induced oxidative responses.

Despite the observed upregulation of VEGF and FGF at the molecular level, AgNP exposure did not result in a statistically significant enhancement of fibroblast migration under the tested conditions. Previous studies have reported that AgNPs can alter cytoskeletal organization and modulate cell migration in fibroblasts and endothelial cells, depending on experimental context (Vieira et al., 2017; He et al., 2024). Fibroblast migration is a critical component of wound healing, contributing to wound closure, extracellular matrix remodeling, and coordination with angiogenic processes (Li & Wang, 2011; Xue & Jackson, 2015; Bi et al., 2019). A non-significant increase in migratory capacity suggests that elevated growth factor expression alone may be insufficient to drive functional repair responses under nanoparticle-induced stress. Nanoparticle exposure can activate stress-response signaling and increase cytokine or growth factor expression without necessarily improving cell function, indicating a disconnect between molecular signaling and functional repair outcomes (Liu & Tang, 2017; Cameron et al., 2022). In the context of oxidative or structural stress, fibroblasts may activate compensatory signaling programs aimed at preserving tissue homeostasis, even when key functional processes such as migration are not significantly enhanced. The intracellular accumulation of AgNPs observed in the present study, particularly within mitochondria and cytoplasmic vesicles, may influence these processes and thereby affect the ability of cells to translate pro-angiogenic signaling into effective motility.

Previous studies have reported that AgNPs can exert modulatory effects on angiogenic signaling, although outcomes vary widely depending on cell type, particle characteristics, and experimental context. For example, AgNPs synthesized from *Nigella sativa* extract promoted wound healing in keratinocytes by enhancing VEGF and PDGF expression (Palanisamy et al., 2024). In contrast, AgNPs suppressed VEGF- and IL-1*β*-induced vascular permeability in porcine retinal endothelial cells *via* inhibition of Src signaling (Sheikpranbabu et al., 2009), demonstrating an inhibitory effect in vascular-specific contexts. Similarly, studies in human skin cells showed that AgNPs reduced VEGF expression in normal human dermal fibroblasts (NHDFs) and epidermal keratinocytes (NHEKs) at 25 ppm (Franková et al., 2016), and that FGF expression in NHDFs was unaffected by 25 µg/mL exposure (Galandáková et al., 2016). In addition, AgNPs have been reported to exert anti-angiogenic effects in endothelial cells by suppressing VEGF-mediated proliferation and migration (Kalishwaralal et al., 2009). Collectively, these findings underscore the context-dependent nature of AgNP-mediated effects on angiogenic signaling. In the present study, NIH/3T3 fibroblasts exposed to standardized AgNPs exhibited a concentration-dependent upregulation of VEGF and FGF protein expression despite clear indicators of cellular stress, including intracellular nanoparticle accumulation, ultrastructural alterations, reduced viability at higher concentrations, and evidence of early oxidative response, although ROS measurements did not reach statistical significance. Importantly, although a modest increase in migration was observed, this did not reach statistical significance, indicating that enhanced growth factor expression did not translate into a measurable functional improvement in migratory capacity. Taken together, these findings support the interpretation that AgNP-induced VEGF and FGF upregulation reflects a stress-associated compensatory response rather than a direct stimulation of functional angiogenic activity.

From a biomaterial perspective, these findings highlight the importance of dose-dependent effects of AgNPs. While low or carefully controlled exposures may modulate signaling pathways relevant to tissue repair, higher concentrations are associated with cytotoxicity and impaired cellular function, potentially limiting their regenerative utility. These results underscore the need for cautious optimization of AgNP incorporation into wound dressings and tissue-engineering scaffolds to balance antimicrobial efficacy with cellular compatibility.

This study has several limitations that should be acknowledged. First, dedicated positive controls for cytotoxicity (*e.g.*, H_2_O_2_) and angiogenic induction (*e.g.*, hypoxia-mimetic agents) were not included. The absence of these reference treatments limits direct benchmarking of the magnitude of AgNP-induced effects against well-established cytotoxic or pro-angiogenic stimuli. Future studies incorporating such controls will be important for better contextualizing the biological relevance of the observed responses. Additional limitations include the use of a single fibroblast cell line and limited exposure time points, which may not fully capture the temporal and cell type-specific variability of fibroblast responses to AgNPs. Measurements were performed at 6- and 24-hour time points in a single murine fibroblast model (NIH/3T3) under serum-free conditions, which may enhance nanoparticle uptake and stress responses and thereby limit the generalizability of the findings. Future work should therefore include multiple time points, additional fibroblast models (including primary human cells), and serum-containing conditions to better approximate physiological environments and verify the robustness of the observed effects. Importantly, the AgNP concentrations used in this study (25–100 µg/mL) are higher than typical systemic exposure levels and may not fully reflect physiological conditions. These concentrations were selected to approximate local exposure at the biomaterial–cell interface, where fibroblasts are in direct contact with AgNP-containing materials. However, future studies should incorporate lower, more physiologically relevant concentrations to better define the therapeutic window and enable more accurate assessment of dose-dependent biological responses.

## Conclusions

This study demonstrates that AgNP exposure induces stress-associated responses in NIH/3T3 fibroblasts, accompanied by upregulation of VEGF and FGF expression. Notably, this molecular response was not paralleled by a significant enhancement in migratory capacity, indicating a disconnect between growth factor expression and functional cellular outcomes. These findings suggest that AgNP-induced VEGF and FGF upregulations reflect a compensatory response to cellular stress rather than functional angiogenic activation. From a biomaterials perspective, this distinction underscores the importance of dose optimization when incorporating AgNPs into regenerative systems, as modulation of signaling pathways may not directly translate into improved cellular function.

##  Supplemental Information

10.7717/peerj.21694/supp-1 Supplemental Information 1 Complete raw datasets supporting all quantitative analyses reported in this study

10.7717/peerj.21694/supp-2 Supplemental Information 2 Uncropped blots

## Additional Information and Declarations

### Data Availability

The following information was supplied regarding data availability: The data is available in the Supplemental Files.
